# Platelet-Rich Plasma for Adhesive Capsulitis: A Systematic Review

**DOI:** 10.7759/cureus.46580

**Published:** 2023-10-06

**Authors:** Erica Blanchard, Jackson Harvi, John Vasudevan, Randel L Swanson

**Affiliations:** 1 Physical Medicine and Rehabilitation, University of Pennsylvania Perelman School of Medicine, Philadelphia, USA; 2 Physical Medicine and Rehabilitation, Philadelphia College of Osteopathic Medicine, Philadelphia, USA; 3 Center for Neurotrauma, Neurodegeneration and Restoration, Corporal Michael J. Crescenz VA Medical Center, Philadelphia, USA

**Keywords:** regenerative medicine, shoulder joint, platelet rich plasma, frozen shoulder, adhesive capsulitis

## Abstract

Adhesive capsulitis (AC) is a common cause of shoulder pain seen in 3%-5% of the population. Platelet-rich plasma (PRP) is platelet-rich blood with pro-inflammatory and anti-inflammatory properties that has been proposed as a treatment option for patients with AC. The purpose of this study was to analyze outcomes of range of motion (ROM) and subjective outcomes, including the visual analog scale (VAS), disability of arm, shoulder, and hand (DASH), and shoulder pain and disability index (SPADI) scores.

PubMed, Embase, and Cochrane databases were searched, and manuscripts were screened using defined preferred reporting items for systematic reviews and meta-analyses (PRISMA) criteria. Two reviewers independently screened articles for inclusion/exclusion using PICOS criteria and extracted data regarding ROM and subjective outcome scores. Nineteen total articles were included.

Eleven of the 19 studies recorded ROM as a dependent variable. All articles reported improved ROM with PRP injection when compared to baseline. When recording degrees of shoulder ROM in different planes at the latest follow-up, there were a total of 67 comparative data points for PRP vs. control. Of the 67 comparisons, 62 (93%) had a larger final ROM in the PRP group. VAS scores were reported in 16 of the 19 studies, DASH scores were reported in eight of the 19 articles, and SPADI scores were reported in seven of the 19 articles. VAS, DASH, and SPADI scores were all superior in the PRP group compared to the control. Two studies reported the same final VAS score, but the PRP groups had a larger overall improvement.

Of the studies that reported objective ROM outcomes, the PRP group had greater ROM at the longest follow-up compared to control in the vast majority of comparisons. For the studies that reported subjective outcomes, all patients that received PRP had a decrease in VAS pain scores and an improvement in DASH and SPADI questionnaires.

## Introduction and background

Adhesive capsulitis (AC) is a common cause of shoulder pain seen in 3%-5% of the population, and the condition is characterized by adhesions and fibrosis of the glenohumeral capsule, which leads to pain and restriction in active and passive ROM [1]. The history of AC started in 1845, when Duplay recognized chronic shoulder pain and classified it as “scapulohumeral periarthritis” [2]. In 1934, Codman coined the term “frozen shoulder,” which was characterized by debilitating loss of shoulder motion, and described this condition as “difficult to define, difficult to treat, and difficult to explain from the point of view of pathology” [3].

On a cellular level, for AC, there is inflammation leading to capsular hyperplasia and an imbalance of fibrosis and collagen remodeling [4]. AC is associated with a proinflammatory phase characterized by increased Interleukin (IL)-1a, IL-1b, IL-6, IL-8, Tumor Necrosis Factor (TNF)-alpha, Cyclo-Oxygenase (COX)-1, COX-2, mitogen-activated protein kinases, nuclear factor (NF) kappa B, CD29, transforming growth factor (TGF)-beta, and vascular endothelial growth factor (VEGF) [4]. With this inflammation, there is a cascade of neo-angiogenesis, chondrogenesis, and neoinnervation in the contracted capsule of the shoulder [4]. However, the full etiology of AC is complex and remains incompletely understood. It is thought that there is a fundamental imbalance of matrix synthesis and degradation leading to impaired matrix remodeling, which then leads to the propagation of fibroblasts that release type I and type III collagen [5]. Anatomically, this leads to capsular hyperplasia and fibrosis that reduce capsular volume and limit movements of the glenohumeral joint [6]. 

Therapeutic exercises, particularly joint mobilization and stretching, are the mainstay of managing AC [7]. These exercises are frequently advised along with various pain-relieving agents, such as oral or injectable medications [7]. Several injections, including intra-articular corticosteroids (CS), sodium hyaluronate, and hydraulic distension (hydro-dilatation), have been advocated to reduce shoulder pain [7]. Other less common options can include ultrasound therapy to augment blood flow and re-vascularization, surgical manipulation under anesthesia, and cryotherapy [4]. Regenerative medicine using orthobiologic agents such as PRP (platelet-rich plasma) has recently been gaining interest as an emerging therapeutic option.

PRP is an autologous concentration of human platelets with a combination of pro-inflammatory, anti-inflammatory, anti-nociceptive, and regenerative properties [8]. PRP leads to the release of growth factors (e.g., platelet-derived growth factor, transforming growth factor-β, vascular, and epidermal endothelial growth factor), which contribute to the pro-inflammatory effects. The anti-inflammatory effects work by decreasing leukocyte recruitment and releasing anti-inflammatory factors like hepatocyte growth factor, tumor necrosis factor, and vasoactive intestinal peptide [5]. The nociceptive effect is thought to be due to an augmentation of cannabinoid receptors [9]. In terms of clinical application, researchers have shown the positive effects of PRP in several other disorders, including chronic elbow tendinopathy [10], chronic Achilles tendinopathy [11], knee osteoarthritis [12], and rotator cuff tendon tears [13]. 

It is important to mention corticosteroid injections since they were commonly used as the control treatment in many of the reported studies in this systematic review. Intra-articular corticosteroids are commonly used because they are cost-effective, shown to provide pain relief, and also limit the development of capsular fibrosis with anti-inflammatory effects [14]. However, some side effects of corticosteroids include the possibility of tendon rupture, hyperglycemia, skin depigmentation, joint infection, periarticular calcifications, and subcutaneous atrophy [15,16]. Corticosteroid exposure on a systemic level can also cause bone mass loss by altering the fragile balance between osteoclast and osteoblast activity, thus increasing the risk of fractures [17]. There are no reported side effects of PRP besides pain at the injection site. Its autologous nature prevents an immunological reaction and offers good therapeutic safety [18].

The objective of this systematic review was to analyze the literature that is published on the application of PRP for AC. The demographics of the patients, PRP characteristics, and the different types of control groups were also evaluated. The outcomes evaluated were objective values of range of motion (ROM) and subjective scores, including the visual analogue scale (VAS), shoulder pain and disability index (SPADI), and disabilities of the arm, shoulder, and hand score (DASH) [19].

## Review

Methods

Search Strategy

A PubMed, Embase, and Cochrane search of all articles from any date through September 6th, 2022, returned 345 unique results using the combined keywords ((Adhesive Capsulitis OR Shoulder Joint OR Frozen Shoulder OR Periarthritis) AND (Platelet Rich Plasma)).

Eligibility Criteria 

Studies were included if they met the eligibility criteria established according to the participant, comparisons, outcomes, and study designs (PICOS) framework [20].

Participants

Participants had to be human subjects of adult age or sex. Patients must have been diagnosed with AC. This diagnosis could have been the first diagnosis of AC or could be a recurrent AC diagnosis if the patient was actively having symptoms during the study intervention. Participants were included regardless of clinic or hospital setting. 

Interventions

PRP must be injected into a shoulder for the treatment arm of the studies included. Other interventions in addition to PRP were acceptable, such as patients also receiving physical therapy or taking NSAIDS, as long as the control group also received this intervention. Any type of PRP preparation or injection regimen was allowed as long as at least one or more injections of PRP were performed. 

Comparisons

The comparison or control groups could include any other intervention that is considered standard of care for AC. Some examples include medication management alone (e.g., NSAIDs), ROM exercises at home or with physical therapy, hydro-dissection with saline, or corticosteroid injection(s). Comparison groups were not required for study inclusion.

Outcomes

The objective outcome analyzed was ROM. Other subjective outcomes included pain scores such as the VAS, SPADI, and DASH.

Exclusion

Studies were excluded if they were not using human subjects, if the patients did not have a diagnosis of AC, if the treatment arm of patients was not injected with PRP, and if the article was a review article, systematic review, or meta-analysis. 

Study Screening

**Figure 1:**
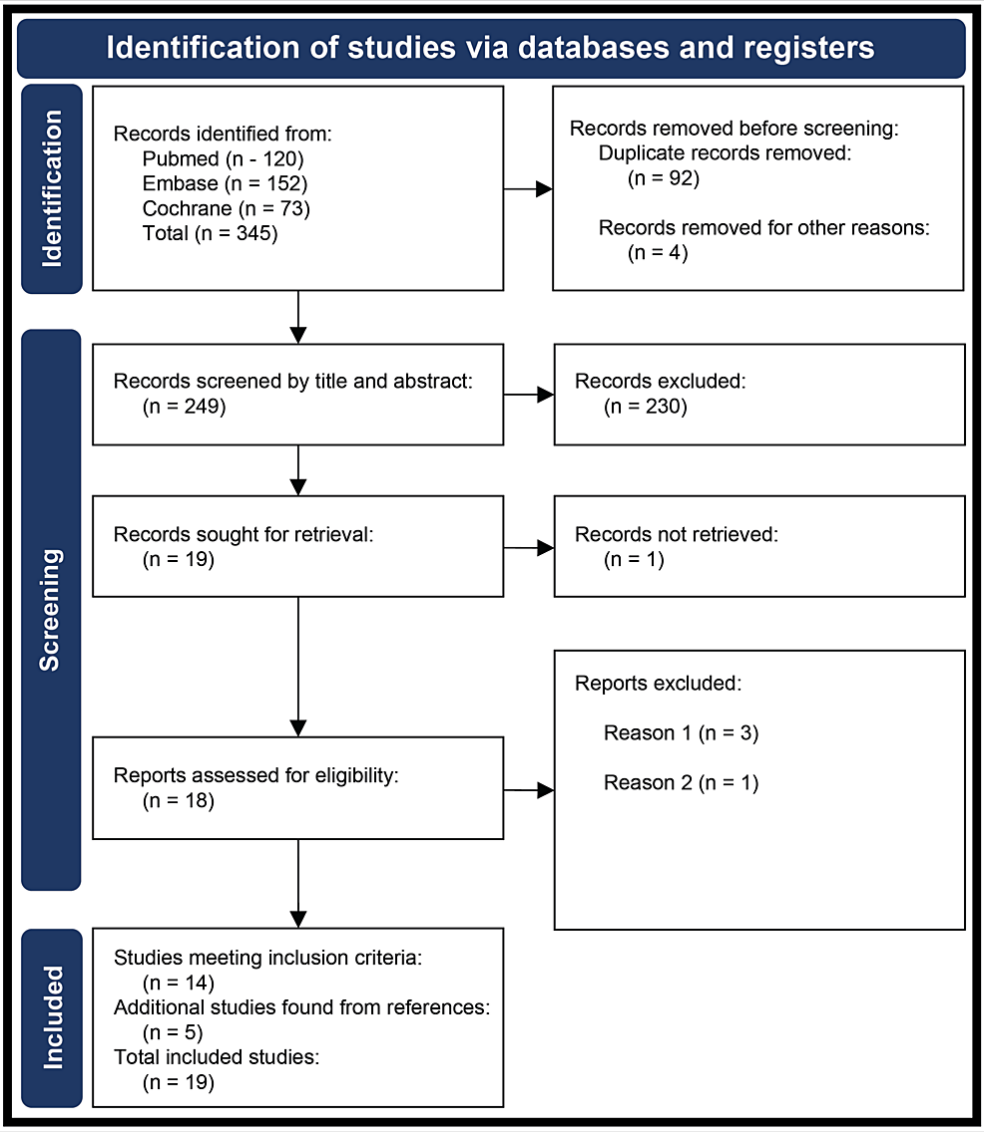
Preferred reporting items for systematic reviews and meta-analyses (PRISMA) flow diagram showing the selection process of reviewed articles Defined reasons reports were excluded: 1) review article, systematic review, or meta-analysis; 2) letter to editor.

Articles were initially screened based on their titles and abstracts. Two reviewers independently screened the retrieved articles based on the defined PICOS criteria detailed above to create the final list of articles to be included in the review. Screening discrepancies were resolved through a third reviewer. A detailed description of the article selection process is shown in the preferred reporting items for systematic reviews and meta-analyses (PRISMA) flow diagram (Figure 1).

Data Abstraction

Two reviewers independently abstracted relevant data from the included articles and recorded the data in a predesigned spreadsheet. Details extracted from the articles included title, author, type of study, year of publication, PRP characteristics, control intervention, and outcomes. Outcomes were related to study purpose, conclusions, results, follow-up time, VAS, DASH, SPADI, and ROM. 

Results

Study Characteristics

A total of 19 articles were found to meet our PICOS criteria and were a part of this review, including 10 randomized controlled trials (RCT), two case series, six cohort studies, and one case report (Table 1) [4,6-8,16-18,21-32]. The outcomes that were reported in each article can be seen in Table 1. The duration of follow-up across these studies ranged from 4 weeks to 48 weeks. The total number of patients across the 19 articles receiving PRP was 592, and the total number of patients in control groups was 671. The demographics of patients, specifically average age and gender breakdown, are reported in Table 2. The method of PRP preparation, including how the PRP sample was spun, and any further description of type of PRP are presented in Table 3.

**Table 1:** Descriptive details of the included articles RCT: Randomized controlled trial; PRP: Platelet-rich plasma; SGB: Stellate ganglion block; US: Ultrasound; CS: Corticosteroid; PT: Physical therapy

Study Reference	Title	Study Type	Study Length (weeks)	PRP Injection #	Volume PRP Injection (mL)	Comparison	Follow-up Intervals (weeks)	Outcomes
Jeyaraman et al., 2018 [4]	The comparative and prospective study on efficacy and functional outcome of autologous platelet rich plasma injection vs hydrodissection in adhesive capsulitis of shoulder	Prosp Cohort	48	1	3	Hydrodissection	4,24,48	PRP injection is considered superior to hydrodissection, as platelet-rich plasma injection provides growth factors for tissue rejuvenation, while hydrodissection leads to forced capsular rupture. The group who received PRP therapy showed better pain relief, functional range of movements, and improved quality of life than the group who received hydrodissection.
Aslani et al., 2016 [6]	Platelet-Rich Plasma for Frozen Shoulder: A Case Report	Case Report	n/a	2	n/a	none	n/a	PRP decreases pain and increases upper-limb function. Also, it can improve shoulder ROM in various directions.
Kothari et al., 2017 [7]	Comparative Efficacy of Platelet Rich Plasma Injection, Corticosteroid Injection and Ultrasonic Therapy in the Treatment of Periarthritis Shoulder	RCT	12	1	2	2cc methylprednisolone injection or US therapy	3,6,12	Single injection of PRP is effective and better than corticosteroid injection or ultrasonic therapy.
Barman et al., 2019 [8]	Single Intra-articular Platelet-Rich Plasma Versus Corticosteroid Injections in the Treatment of Adhesive Capsulitis of the Shoulder A Cohort Study	Prosp Cohort	12	1	4	2cc methylprednisolone + 2cc of 2% Lidocaine injection	3,6,12	Single dose of PRP injection was found to be more effective than a CS injection in terms of improving pain, disability, and shoulder range of movement.
Yadav et al., 2021 [16]	Comparative Assessment of the Significance of Platelet Rich Plasma (PRP) and Corticosteroid Injection in Management of Adhesive Capsulitis of Shoulder	Prospective Comparative	12	1	n/a	2cc methylprednisolone injection	1,4,12	PRP is not inferior to CS injection in any of the measured parameters, and both groups experienced similar benefits with no statistical differences detected in ROM or VAS scores.
Lee et al., 2021 [17]	Allogenic Pure Platelet-Rich Plasma Therapy for Adhesive Capsulitis A Bed-to-Bench Study With Propensity Score Matching Using a Corticosteroid Control Group	Prosp Cohort	24	1	n/a	Triamcinolone injection	1,4,12,24	Pure PRP significantly relieved pain and improved ROM, shoulder strength, and function in a manner comparable with that of corticosteroid treatment. The corticosteroid group showed faster improvements in pain and shoulder function that did not last long, whereas the PRP group showed a slower recovery but steadier healing effects.
Gupta et al., 2022 [18]	Comparison of the Efficacy of Platelet-Rich Plasma (PRP) and Local Corticosteroid Injection in Periarthritis Shoulder: A Prospective, Randomized, Open, Blinded End-Point (PROBE) Study	Prospective Randomized	24	1	2	2cc triamcinolone (40mg/mL) injection	3,12,24	PRP and triamcinolone injections are effective in reducing pain and disability scores in terms of VAS and DASH scores. The triamcinolone group showed a better effect in short-term outcomes (12th-week analysis), whereas PRP showed better results in long-term outcomes (24th-week analysis).
Agrawal et al., 2019 [21]	Management of Adhesive Capsulitis of Shoulder Joint by Single Platelet Rich Plasma Injection	Prosp Cohort	4	1	4	none	0.4,4	PRP treatment showed an increase in pain for a few participants on the third day, causing a decreased active range of motion. However, PRP resulted in significant improvement in the mean active range of shoulder abduction, flexion, external rotation, and internal rotation in the 1-month follow-up. This study demonstrates that a single injection of PRP improves both pain and the ROM of the shoulder joint.
Aslani et al., 2020 [22]	Clinical Results of Platelet-Rich Plasma in Frozen Shoulder	Case Series	25	2	n/a	none	4,6,25	Demonstrated clinically and statistically significant improvement in patients' pain and disability outcomes following PRP injection.
Barman, et al., 2021 [23]	The benefit of platelet-rich plasma injection over institution-based physical therapy program in adhesive capsulitis patients with diabetes mellitus: prospective observational cohort study	Prosp Cohort	12	1	4	Physical therapy	3,6,12	PRP injections significantly improved shoulder pain and function compared with an institution-based PT program in a diabetic population.
Calis et al., 2019 [24]	Effects of Platelet-rich Plasma Injection on Adhesive Capsulitis: An Interventional Case Series	Case Series	12	2	n/a	none	2,6,12	Significant improvements were detected in VAS and SPADI scores when compared with baseline (p<0.05). There was a significant improvement in active and passive ROMs when compared with baseline (p<0.05).
Ghaffar et al., 2019 [25]	Combined platelet rich plasma intra-articular shoulder injection and stellate ganglion block. A new technique for management of chronic post-mastectomy shoulder pain syndrome	RCT	12	3	n/a	Group A: SGB (ketamine+5cc bupivacaine) + PRP. Group B: SGB + 10cc saline	4,8,12	Patients who received combined PRP and SGB with ketamine and bupivacaine showed statistically significant improvement regarding pain with shoulder movements, ROM, and DASH scores. The combination provides dramatic improvement in post-mastectomy chronic shoulder pain.
Lin, 2018 [26]	Platelet-rich plasma injection in the treatment of frozen shoulder: A randomized controlled trial with 6-month follow-up	RCT	12	3	n/a	1% Procaine+25mg Hydrocortisone injection + PT	1,4,12	PRP was superior to and longer than procaine. Injection with PRP was beneficial to reduce the pain of a frozen shoulder and to improve shoulder function with good tolerance.
Karabaş et al., 2021 [27]	Effects of platelet-rich plasma injection on pain, range of motion, and disability in adhesive capsulitis: A prospective, randomized-controlled study	RCT	12	2	3	Home exercise	2,6,12	Addition of PRP to exercise treatment can improve patients' joint mobility but not pain or disability in patients with adhesive capsulitis. Greater improvement in terms of ROM scores, compared to the control group, while the VAS and SPADI scores were similar to the control group.
Kumar et al., 2021 [28]	Randomized controlled trial of functional outcome of periarthritis of shoulder (Adhesive Capsulitis) in a group of 60 patients using intraarticular triamcinolone vs. intraarticular platelet rich plasma	RCT	24	1	n/a	2-3cc methylprednisolone + 1% Lidocaine injection	4,8,12,16,20,24	PRP treatment for 24 weeks resulted in significant improvement in range of shoulder motion, pain, and function compared to triamcinolone.
Shahzad et al., 2021 [29]	Comparison of Functional Outcome Between Intra-Articular Injection of Corticosteroid Versus Platelet-Rich Plasma in Frozen Shoulder: A Randomized Controlled Trial	RCT	12	1	2	2cc methylprednisolone injection	12	Intra-articular injection of PRP resulted in a substantial improvement in the VAS score, UCLA, and ROM when compared to intra-articular corticosteroid injection.
Thu et al., 2020 [30]	Comparison of ultrasound-guided platelet-rich plasma injection and conventional physical therapy for management of adhesive capsulitis: a randomized trial	RCT	6	1	4	Physical therapy	1,3,6	PRP had a comparable effect on pain reduction, functional improvement, and shoulder joint ROM improvement compared with PT. There was no significant difference in the measured outcomes between the two groups. However, there was less acetaminophen consumption after PRP injection compared with that after PT.
Unlu et al., 2021 [31]	Efficacy of platelet-rich plasma injections in patients with adhesive capsulitis of the shoulder	RCT	12	3	2	Saline injection	4,8,12	PRP group had better results in both pain and disability in the first and third months when compared to the placebo group.
Upadhyay et al., 2020 [32]	Ongoing Efficacy of Platelet-rich Plasma vs Corticosteroid Injection in Patients with Adhesive Capsulitis: A Prospective Randomized Assessor-blind Comparative Analysis	RCT	24	1	2	Triamcinolone injection	4,12,24	PRP injections promote healing and demonstrate a more linear and sustained improvement both in pain scores and disability scores as compared to steroid injections.

**Table 2:** Demographic data of included articles *: Reported as mean age.

Study Reference	n	Age of PRP Group*	Age of Control Group*	# Males/Females in PRP Group	# Males/Females in Comparison Group
Jeyaraman et al., 2018 [4]	91	52	57	29/17	32/13
Aslani et al., 2016 [6]	1	45	n/a	1/0	n/a
Kothari et al., 2017 [7]	180	52	52	34/28	52/66
Barman et al., 2019 [8]	55	50	50	12/16	10/17
Yadav et al., 2021 [16]	60	50	50	n/a	n/a
Lee et al., 2021 [17]	30	60	58	7/8	5/10
Gupta et al., 2022 [18]	60	48	47	12/18	13/17
Agrawal et al., 2019 [21]	20	50	50	n/a	n/a
Aslani et al., 2020 [22]	44	52	n/a	10/34	n/a
Barman et al., 2021 [23]	70	49	50	14/21	15/20
Calis et al., 2019 [24]	9	54	54	2/7	n/a
Ghaffar et al., 2019 [25]	64	55	51	0/32	0/32
Lin, 2018 [26]	61	60	58	9/21	11/19
Karabaş et al., 2021 [27]	40	57	57	14/6	7/13
Kumar et al., 2021 [28]	60	55	57	8/22	16/14
Shahzad et al., 2021 [29]	202	70	57	43/59	41/59
Thu et al., 2020 [30]	64	53	57	4/27	9/21
Unlu et al., 2021 [31]	32	57	57	6/11	5/10
Upadhyay et al., 2020 [32]	120	48	46	25/35	25/35

**Table 3:** Details of platelet-rich plasma (PRP) preparations LP: Leukocyte-poor; LR: Leukocyte-rich; PRP: Platelet-rich plasma

Study Reference	PRP System Used	Type of PRP Used	Spin Technique
Jeyaraman et al., 2018 [4]	Manual method	Not specified	1st: 3,000 rpm x 10 minutes 2nd: 5,000 rpm x 10 minutes
Aslani et al., 2016 [6]	Arthrex-ACP system	Not specified	5,000 rpm x 5 minutes
Kothari et al., 2017 [7]	Not specified	Not specified	Not specified
Barman et al., 2019 [8]	Manual method	LP PRP	1,800 rpm x 14 minutes
Yadav et al., 2021 [16]	Manual method	Not specified	1st: 1,500 rpm x 6 minutes 2nd: 3,400 rpm x 15 minutes
Lee et al., 2021 [17]	Not specified	Not specified	Not specified
Gupta et al., 2022 [18]	Manual method	Not specified	1st: 1,500 rpm x 15 minutes 2nd: 2,500 rpm x 10 minutes
Agrawal et al., 2019 [21]	Manual method	LR PRP	Not specified
Aslani et al., 2020 [22]	Arthrex-ACP system	Not specified	1,500 rpm x 5 minutes
Barman et al., 2021 [23]	Plasmamed PRP kit	LP PRP	1,800 rpm x 14 minutes
Calis et al., 2019 [24]	Manual method	Not specified	1st: 1,195 rpm x 20 minutes 2nd: 1,890 rpm x 15 minutes
Ghaffar et al., 2019 [25]	Manual method	LR PRP	Not specified
Lin, 2018 [26]	Manual method	Not specified	1st: 2,000 rpm x 10 minutes 2nd: 2,800 rpm x 7 minutes
Karabaş et al., 2021 [27]	Manual method	Not specified	1st: 1,195 rpm x 20 minutes 2nd: 1,890 rpm x 15 minutes
Kumar et al., 2021 [28]	Manual method	Not specified	1st: 1,800 rpm x 8 minutes. 2nd: 2,600 rpm x 8 minutes
Shahzad et al., 2021 [29]	Not specified	Not specified	Not specified
Thu et al., 2020 [30]	Manson Power PRP tube	Not specified	1,500 rpm x 8 minutes
Unlu et al., 2021 [31]	Neotec Easy PRP kit	Not specified	1st: 1,200 rpm x 5 minutes 2nd: 1,200 rpm x 10 minutes
Upadhyay et al., 2020 [32]	Manual method	LP PRP	1,500 rpm x 15 minutes

When analyzing the articles in this study, 10 of the articles used a corticosteroid injection as the control. Barman et al. [8] compared one injection of PRP to an injection of Methylprednisolone 2cc + 2cc of 2% Lidocaine. Ghaffar et al. [25] compared three injections of PRP + Stellate Ganglion Block + Ketamine + 5 cc Bupivacaine 0.5% + 10 cc Saline to the same regimen without PRP. Kothari et al. [7] looked at one injection of PRP with either an injection of 2 cc methylprednisolone or ultrasound therapy. Gupta et al. [18], Lee et al. [17], and Upadhyay et al. [32] compared one injection of PRP to a triamcinolone injection. Lin et al. [26] compared three PRP injections to 1% procaine + 25mg hydrocortisone injection + PT. Shahzad et al. [29] and Yadav et al. [16] both compared one PRP injection to a 2 cc methylprednisolone injection. Kumar et al. [28] compared one injection of PRP to one injection of 2-3 cc Methylprednisolone + 1% Lidocaine.

Three studies used PT, or home exercises, as the control [23,27,30]. Two studies [4,31] evaluated saline hydrodissection vs. PRP. Finally, four studies [6,21,22,24] evaluated the outcomes of PRP without direct comparison.

Range of Motion

ROM data can be found in Tables 4-8. Eleven of the 19 studies recorded ROM as a dependent variable. Ten of the 11 studies recorded active ROM, one recorded passive, and five recorded both active and passive. In terms of outcomes, all 11 articles reported improved ROM with PRP injection when compared to baseline. All articles except one also reported improved ROM in the control groups when compared to baseline. For the control groups, the only ROM that did not improve was in Kothari et al. [7], where external rotation in the ultrasound comparison group did not change when compared to baseline. This was also the only article that had two control groups, one group being an injection of 2 cc Methylprednisolone and another group being ultrasound. Across the 11 articles, there were a total of 67 ROM comparisons of PRP to control, and 62 of the 67 (93%) had a greater ROM value at the longest follow-up, three instances had equivalent values at the latest follow-up, and in two instances, the control had greater ROM values at the longest follow-up. 

**Table 4:** Range of motion data, average degrees of active and passive flexion All range of motion values are reported as degrees, unless otherwise indicated. *: reported as overall percent (%) improvement; ***: data for Kothari et al. [7] ultrasound control group. PRP: Platelet-rich plasma.

Study Reference	ACTIVE Pre-PRP	ACTIVE Post-PRP	ACTIVE Pre-Control	ACTIVE Post-Control	PASSIVE Pre-PRP	PASSIVE Post-PRP	PASSIVE Pre-Control	PASSIVE Post-Control
Jeyaraman et al., 2018 [4]	70	130	45	120	-	-	-	-
Aslani et al., 2016 [6]	70	150	-	-	-	-	-	-
Kothari et al., 2017 [7]	96	146	97	133	102	151	103	138
***Kothari et al., 2017 [7]	-	-	97	125	-	-	103	129
Barman et al., 2019 [8]	78	128	75	119	99	149	100	144
Lee et al., 2021 [17]	121	158	117	157	-	-	-	-
Agrawal et al., 2019 [21]	-	75%*	-	-	-	-	-	-
Aslani et al., 2020 [22]	65	148	-	-	-	-	-	-
Barman et al., 2021 [23]	81	130	78	106	98	148	95	125
Ghaffar et al., 2019 [25]	91	146	94	123	-	-	-	-
Karabaş et al., 2021 [27]	94	154	103	134	106	161	110	143
Shahzad et al., 2021 [29]	83	155	84	127	-	-	-	-
Thu et al., 2020 [30]	-	-	-	-	105	146	102	142
Unlu et al., 2021 [31]	105	168	108	133	123	175	124	146

**Table 5:** Range of motion data, average degrees of active and passive extension ***: data for Kothari et al. [7] ultrasound control group. PRP: Platelet-rich plasma.

Study Reference	ACTIVE Pre-PRP	ACTIVE Post-PRP	ACTIVE Pre-Control	ACTIVE Post-Control	PASSIVE Pre-PRP	PASSIVE Post-PRP	PASSIVE Pre-Control	PASSIVE Post-Control
Jeyaraman et al., 2018 [4]	30	50	30	50	-	-	-	-
Kothari et al., 2017 [7]	36	-	32	-	42	-	37	-
***Kothari et al., 2017 [7]	-	-	29	-	-	-	32	-
Barman et al., 2019 [8]	22	39	20	36	29	46	29	46
Barman et al., 2021 [23]	22	39	21	34	29	46	28	41
Ghaffar et al., 2019 [25]	36	68	36	62	-	-	-	-
Karabaş et al., 2021 [27]	43	58	44	55	46	60	47	57

**Table 6:** Range of motion data, average degrees of active and passive abduction ***: data for Kothari et al. [7] ultrasound control group. PRP: Platelet-rich plasma.

Study Reference	ACTIVE Pre-PRP	ACTIVE Post-PRP	ACTIVE Pre-Control	ACTIVE Post-Control	PASSIVE Pre-PRP	PASSIVE Post-PRP	PASSIVE Pre-Control	PASSIVE Post-Control
Jeyaraman et al., 2018 [4]	60	165	50	145	-	-	-	-
Aslani et al., 2016 [6]	75	135	-	-	-	-	-	-
Kothari et al., 2017 [7]	90	142	91	130	96	148	96	136
***Kothari et al., 2017 [7]	-	-	88	117	-	-	96	124
Barman et al., 2019 [8]	68	121	62	101	88	138	89	128
Lee et al., 2021 [17]	104	156	106	159	-	-	-	-
Aslani et al., 2020 [22]	70	140	-	-	-	-	-	-
Barman et al., 2021 [23]	68	119	65	91	88	138	86	114
Ghaffar et al., 2019 [25]	98	148	92	116	-	-	-	-
Karabaş et al., 2021 [27]	78	153	87	126	86	162	94	138
Shahzad et al., 2021 [29]	65	147	65	129	-	-	-	-
Thu et al., 2020 [30]	-	-	-	-	93	132	90	130
Unlu et al., 2021 [31]	76	159	87	119	87	168	100	134

**Table 7:** Range of motion data, average degrees of active and passive internal rotation ***: data for Kothari et al. [7] ultrasound control group. PRP: Platelet-rich plasma.

Study Reference	ACTIVE Pre-PRP	ACTIVE Post-PRP	ACTIVE Pre-Control	ACTIVE Post-Control	PASSIVE Pre-PRP	PASSIVE Post-PRP	PASSIVE Pre-Control	PASSIVE Post-Control
Jeyaraman et al., 2018 [4]	30	60	20	55	-	-	-	-
Kothari et al., 2017 [7]	22	58	22	50	26	60	27	54
***Kothari et al., 2017 [7]	-	-	21	46	-	-	28	49
Barman et al., 2019 [8]	16	48	16	38	26	62	27	53
Lee et al., 2021 [17]	5	10	4	9	-	-	-	-
Barman et al., 2021 [23]	17	48	18	35	25	62	27	48
Ghaffar et al., 2019 [25]	52	78	58	69	-	-	-	-
Karabaş et al., 2021 [27]	30	74	35	68	33	77	38	70
Shahzad et al., 2021 [29]	28	59	28	49	-	-	-	-
Unlu et al., 2021 [31]	45	66	41	51	55	69	50	60

**Table 8:** Range of motion data, average degrees of active and passive external rotation ***: data for Kothari et al. [7] ultrasound control group. PRP: Platelet-rich plasma.

Study Reference	ACTIVE Pre-PRP	ACTIVE Post-PRP	ACTIVE Pre-Control	ACTIVE Post-Control	PASSIVE Pre-PRP	PASSIVE Post-PRP	PASSIVE Pre-Control	PASSIVE Post-Control
Jeyaraman et al., 2018 [4]	30	70	30	60	-	-	-	-
Aslani et al., 2016 [6]	25	35	-	-	-	-	-	-
Kothari et al., 2017 [7]	35	80	34	71	38	86	38	77
***Kothari et al., 2017 [7]	-	-	65	65	-	-	70	38
Barman et al., 2019 [8]	19	48	20	45	25	60	28	54
Lee et al., 2021 [17]	27	37	26	39	-	-	-	-
Aslani et al., 2020 [22]	22	48	-	-	-	-	-	-
Barman et al., 2021 [23]	18	50	19	39	25	63	26	47
Ghaffar et al., 2019 [25]	42	73	37	73	-	-	-	-
Karabaş et al., 2021 [27]	41	82	47	72	45	84	50	76
Shahzad et al., 2021 [29]	38	72	38	56	-	-	-	-
Thu et al., 2020 [30]	-	-	-	-	56	81	53	79
Unlu et al., 2021 [31]	29	75	32	48	37	80	39	54

Subjective Pain Scores

Visual analog scale (VAS) [19] data can be found in Table 9. Sixteen of the 19 studies recorded VAS as a dependent variable. VAS is a patient-reported pain rating scale that has been used as a valid and effective way to assess patient symptoms. This score is usually on a scale of 1-10, with 10 being the worst pain. All articles recorded a markedly decreased VAS after PRP injection when compared to baseline. Control groups in all articles also had a decrease in VAS. When PRP VAS scores were compared to control across 13 articles, all the VAS scores were lower in the PRP group at the furthest follow-up, except two scores were the same as control but with a larger decrease in the PRP group from baseline.

**Table 9:** Mean visual analogue scale (VAS) scores pre- and post-treatment All values are reported as mean, unless otherwise indicated. *: reported as overall percent (%) improvement; **Reported on a scale of 0-100 instead of 1-10; ***: data for Kothari et al. [7] ultrasound control group. PRP: Platelet-rich plasma; VAS: Visual analog scale.

Study Reference	Pre-PRP	Post-PRP	Pre-Control	Post-Control
Jeyaraman et al., 2018 [4]	8.98	2.11	9.18	3.93
Kothari et al., 2017 [7]	8.4	1.9	8.6	3.4
***Kothari et al., 2017 [7]	-	-	8.9	4.5
Barman et al., 2019 [8]	74.28**	15.89**	71.48**	22.77**
Yadav et al., 2021 [16]	8.86	3.22	8.58	3.22
Lee et al., 2021 [17]	6.3	1.1	5.7	2
Gupta et al., 2022 [18]	67.4**	14.33**	69.63**	31.63**
Agrawal et al., 2019 [21]	-	73.3%*	-	-
Aslani et al., 2020 [22]	8.41	2.79	-	-
Barman et al., 2021 [23]	73.1**	15.9**	74.4**	32.6**
Calis et al., 2019 [24]	9	3.5	-	-
Ghaffar et al., 2019 [25]	7.5	2	-	-
Lin, 2018 [26]	7.25	1.95	7.01	4.11
Karabaş et al., 2021 [27]	10	2	9	2
Shahzad et al., 2021 [29]	8.9	0.85	9.5	2.3
Thu et al., 2020 [30]	83	28	83	31
Unlu et al., 2021 [31]	3.2	0.17	3.9	2
Upadhyay et al., 2020 [32]	21	1.35	20.48	14.68

Subjective Outcome Scores

DASH [19] scores were reported in eight of the 19 articles, and results are in Table 10. This is a 30-question form focused on function, and scores range from 0-100, with higher scores indicating a higher disability. All articles showed improvement with both PRP and control interventions. When comparing PRP to control across six studies, there was a lower score at the latest follow-up in all the PRP groups. 

**Table 10:** Mean disabilities of the arm, shoulder, and hand (DASH) scores pre- and post-treatment ***: data for Kothari et al. [7] ultrasound control group. DASH: Disabilities of the arm, shoulder, and hand; PRP: Platelet-rich plasma

Study Reference	Pre-PRP	Post-PRP	Pre-Control	Post-Control
Jeyaraman et al., 2018 [4]	77.91	30.2	78.08	32.28
Kothari et al., 2017 [7]	83.5	18.7	85.7	34
***Kothari et al., 2017 [7]	-	-	88.6	45.2
Lee et al., 2021 [17]	35.4	12.3	37.8	13.1
Gupta et al., 2022 [18]	77.63	18.08	75.36	31.76
Aslani et al., 2020 [22]	65.9	31.9	-	-
Ghaffar et al., 2019 [25]	62	32	62	49
Thu et al., 2020 [30]	53	14	54	20

SPADI [19] scores were reported in seven of the 19 articles, and the results are in Table 11. This is a questionnaire with five questions focused on pain and eight focused on disability, with a total score ranging from 0-100, with higher values indicating a higher disability. All articles showed improvement with both PRP and control. When comparing PRP to control across six studies, a lower score was reported at the latest follow-up for the PRP groups. All outcome scores reported by each article can be found in Table 12.

**Table 11:** Mean shoulder pain and disability index (SPADI) scores pre- and post-treatment PRP: Platelet-rich plasma; SPADI: Shoulder pain and disability index.

Study Reference	Pre-PRP	Post-PRP	Pre-Control	Post-Control
Barman et al., 2019 [8]	69.39	14.32	64.49	18.72
Lee et al., 2021 [17]	52.8	13.3	54.1	17.3
Barman, et al., 2021 [23]	71.1	14.9	69.3	35.6
Calis et al., 2019 [24]	45	15	-	-
Karabaş et al., 2021 [27]	86.2	17.3	78.5	23.8
Kumar et al., 2021 [28]	80	6	73	13
Unlu et al., 2021 [31]	99.7	13.5	107.8	64

**Table 12:** Overview of subjective and objective outcomes reported DASH: Disabilities of the arm, shoulder and hand; ROM: Range of Motion; SPADI: Shoulder pain and disability index; VAS: Visual analogue scale; Y: Yes; N: No.

Study Reference	ROM	VAS	DASH	SPADI
Jeyaraman et al., 2018 [4]	Y	Y	Y	N
Aslani et al., 2016 [6]	Y	N	N	N
Kothari et al., 2017 [7]	Y	Y	Y	N
Barman et al., 2019 [8]	Y	Y	N	Y
Yadav et al., 2021 [16]	N	Y	N	N
Lee et al., 2021 [17]	Y	Y	Y	Y
Gupta et al., 2022 [18]	N	Y	Y	N
Agrawal et al., 2019 [21]	Y	Y	N	N
Aslani et al., 2020 [22]	Y	Y	Y	N
Barman et al., 2021 [23]	Y	Y	N	Y
Calis et al., 2019 [24]	N	Y	N	Y
Ghaffar et al., 2019 [25]	Y	Y	Y	N
Lin, 2018 [26]	N	Y	N	N
Karabaş et al., 2021 [27]	Y	Y	N	Y
Kumar et al., 2021 [28]	N	N	N	Y
Shahzad et al., 2021 [29]	Y	Y	Y	N
Thu et al., 2020 [30]	Y	Y	Y	N
Unlu et al., 2021 [31]	Y	Y	Y	Y
Upadhyay et al., 2020 [32]	N	Y	N	N

Discussion

The incidence of AC is 3%-5% in the general population and 10%-20% in the diabetic population [1]. Patients with diabetes report a higher incidence of AC, likely due to poor circulation to the shoulder joint, abnormal collagen repair, and degenerative changes following tissue injury [33]. There are also many underlying conditions that can be seen with AC, such as hypoadrenalism, trauma, fibromatosis, hyperlipidemia, and malignant neoplasms [34]. AC usually develops between the ages of 40 and 70, is more prevalent in females, and has a predilection for the non-dominant shoulder [7]. Clinically, AC can take up to one to three years to resolve, and there is a high recurrence rate of 20%-40% [7]. Treatment is essential because if delayed or management is not consistent, there may be a higher risk of disability, societal cost, and loss of function [22,23]. Regenerative medicine, including PRP, has become an emerging therapeutic option for AC.

PRP initially exhibits pro-inflammatory action, and then, as time elapses, there is a shift toward an anti-inflammatory effect [35]. With its complex nature, the different mechanisms of PRP might have effects on improving and modulating all phases of tissue repair, including the inflammatory, proliferative, and remodeling phases of capsular healing in AC [8]. In preclinical rodent studies, PRP injection into the glenohumeral joint inhibited strong structural changes in the posterior synovial membrane of rodents in an in vivo shoulder contracture model [36]. Injecting concentrated platelets may reduce the inflammation of the synovium and facilitate the natural healing process of the joint capsule, which ultimately will provide a decrease in pain and improved ROM of the shoulder joint [8]. Some studies have even demonstrated that PRP leads to a larger reduction in synovial membrane hyperplasia as compared to corticosteroid injections [8]. 

The above discussion details the current working theory behind PRP for AC; however, there is still limited research on this topic, and there are only two prior systematic reviews on PRP for AC, which were recently published in December 2022 [36,37]. Yu et al. [37] included only four studies with a short follow-up time of three months. Yu et al. [37] concluded that PRP can effectively relieve pain and improve ROM in patients with AC in the short term compared to other non-operative treatments. Harna et al. [36] included 11 articles, many of which were included in this systematic review. Harna et al. [36] concluded that all studies but one, which had equivocal results, depicted better clinical and functional outcomes with PRP injection(s) compared to other conservative methods in reducing pain and improving movement in AC. Unlike the two prior systematic reviews, this systematic review includes a larger number of articles (19 in total) and extracted and analyzed ROM objective data and subjective scores of VAS, SPADI, and DASH. This is the only systematic review to our knowledge that has analyzed ROM data for PRP use in AC.

In terms of the outcomes of this study, 11 studies evaluated ROM. All studies showed improved ROM with targeted interventions, including both PRP and non-PRP control treatments. The one exception was with Kothari et al. [7], which reported no change in ROM with external rotation in the ultrasound control group. Among all the comparisons of PRP to control, 93% (62 of 67) of the ROM comparative outcomes were greater in the PRP group. All studies looking at VAS, DASH, and SPADI noted improvement in scores with both PRP and control. For these subjective outcome scores, when compared to the control, PRP had improved scores at the latest follow-up with all the reported results. Two VAS scores were the same at the latest follow-up for PRP and control, but the PRP had a larger overall decrease in both of these studies. This systematic review found positive outcomes with PRP compared to other modalities of treatment. This was consistent with the outcomes found in the two prior systematic reviews. 

Going forward, it is important for researchers to record both active and passive ROM, as both can be affected by AC [8]. Only five of the 11 studies (Tables 4-8) that recorded ROM analyzed both active and passive ROM. Also, only six of the 11 (Tables 4-8) studies evaluated all planes of ROM. Although flexion, abduction, and external rotation are often limited in AC [17], they often affect all planes of motion, and it would be more comprehensive for articles to evaluate passive and active ROM in all planes. Besides ROM, it is also valuable to continue monitoring pain and functional outcomes with standardized questionnaires such as VAS, SPADI, and DASH.

In terms of the control groups, Table 1 above shows the wide variation in control methods ranging from no intervention to injection of Stellate Ganglion Block + Ketamine + 5cc Bupivacaine 0.5% + 10cc Saline [25]. Multi-faceted control groups can make it hard to directly compare results. The simpler control groups with one injection of methylprednisolone [7,8,16,29] are easier to standardize and repeat. PT is also a practical control but leaves the outcomes open to the number of therapy sessions and knowledge of the physical therapist. There has been new discussion in the field of rehabilitation to move away from simple checklists to measure outcomes and to instead use the rehabilitation treatment specification system (RTSS). This approach may be more beneficial to measure outcomes in a rehab setting that is complex, dynamic, and individualized, but on the other hand, it may make it harder to compare across studies [38]. This systematic review supports that PRP has been effective for AC, and as Table 1 shows, the studies comparing PRP to steroid controls had better outcomes with PRP at the longest follow-up, except for one study [16], which had equivocal results. Additional high-quality clinical studies investigating PRP for AC that demonstrate the superiority of PRP to current standard-of-care investigations are needed to move toward insurance coverage of PRP for AC.

A downside of the studies in this systematic review is the variance in PRP preparation, as seen in Table 3. There are several types of PRP, and the main categories are leukocyte-rich PRP (LR-PRP) and leukocyte-poor PRP (LP-PRP); and the preparation method, such as the centrifuge speed and duration, affects the concentration of platelets that may be present in the PRP sample [39]. All articles except three reported the centrifuge method for PRP preparation, but there were drastic differences in spin technique across the studies. Only five studies in this systematic review specified the type of PRP used (3 LP-PRP and 2 LR-PRP). Most users demand at least a four-fold increase in platelet concentration to be achieved [23]. It is also important to avoid freezing PRP, as it can reduce its efficacy [23]. Other important factors are volume of injection, use of other local anesthetics, and use of activators [31]. Activators can be sodium bicarbonate, which helps increase growth factor release, or thrombin and CaCl2, which assist with growth factor migration [31]. The delivery of PRP can also have effects, such as performing fenestration during the injection to increase the surface area and stimulate bleeding in hypovascular areas [32]. There is variation in preparation methods and kits to make PRP, which makes the standardization of its use variable in studies [39]. Future studies should establish more descriptive protocols regarding PRP preparation, and all future studies should specify how their PRP was prepared. If possible, studies should also send a sample of the generated PRP for analysis to determine its platelet concentration. This would help determine PRP efficacy and make it easier to replicate.

Another important element of the studies included in this systematic review is that only six of the 19 studies had follow-up time periods beyond one year, as seen in Table 1. Clinically, AC presents in three phases [22]. Phase I is the 'freezing' painful phase, which lasts for about 2-9 months. Phase II is the 'frozen' or stiff/adhesive phase, which lasts 4-12 months. Finally, phase III is the 'thawing' or recovery phase, which lasts between one and three years. After this phase, the pain and stiffness gradually disappear, and movement gradually returns to normal or near normal [22]. PRP can increase the recruitment of stem cells, which deliver high concentrations of alpha-granules with active moieties [8]. The modulation of stem cells can help explain its long-lasting effects, which have been reported to last up to two years [25]. Therefore, for adequate capture of the benefits and therapeutic effects of PRP, studies should aim to have at least a 24-month follow-up period, which will also allow for full evaluation of the progression of AC through its disease course.

Limitations

Systematic reviews are limited by the quality of the evidence in the articles included. Of the 19 articles analyzed in this systematic review, only 10 were randomized controlled trials. The rest were smaller studies, including a case report, case studies, or cohort studies. There was a large variability in follow-up duration, ranging from 4 weeks to 48 weeks. Another limitation was the large variation in the PRP preparation. Finally, on a clinical level, none of the studies discussed the cost of PRP. PRP can be expensive [39], ranging from $500 to $2,000, and is often not covered by insurance companies.

## Conclusions

PRP offers a promising treatment option for patients with AC. This systematic review presents 19 original studies that evaluate the outcomes of PRP for AC. Eleven studies reported objective ROM outcomes, and 93% of the comparisons between the PRP treatment arm and control arm had larger ROM in the PRP group at the longest follow-up. For the studies that reported subjective outcomes, all patients that received PRP had a decrease in VAS pain scores and an improvement in SPADI and DASH outcome questionnaires. No adverse effects were reported across any of the studies. Future studies that look at PRP for AC should aim to report active and passive ROM in all planes, report subjective standardized functional and pain scores, use a control group that is easily reproducible, report their PRP preparation methods, and follow outcomes up at least one year out.
